# Numerical Analysis of an *H* ^1^-Galerkin Mixed Finite Element Method for Time Fractional Telegraph Equation

**DOI:** 10.1155/2014/371413

**Published:** 2014-07-24

**Authors:** Jinfeng Wang, Meng Zhao, Min Zhang, Yang Liu, Hong Li

**Affiliations:** ^1^School of Statistics and Mathematics, Inner Mongolia University of Finance and Economics, Hohhot 010070, China; ^2^School of Mathematical Sciences, Inner Mongolia University, Hohhot 010021, China

## Abstract

We discuss and analyze an *H* ^1^-Galerkin mixed finite element (*H* ^1^-GMFE) method to look for the numerical solution of time fractional telegraph equation. We introduce an auxiliary variable to reduce the original equation into lower-order coupled equations and then formulate an *H* ^1^-GMFE scheme with two important variables. We discretize the Caputo time fractional derivatives using the finite difference methods and approximate the spatial direction by applying the *H* ^1^-GMFE method. Based on the discussion on the theoretical error analysis in *L* ^2^-norm for the scalar unknown and its gradient in one dimensional case, we obtain the optimal order of convergence in space-time direction. Further, we also derive the optimal error results for the scalar unknown in *H* ^1^-norm. Moreover, we derive and analyze the stability of *H* ^1^-GMFE scheme and give the results of a priori error estimates in two- or three-dimensional cases. In order to verify our theoretical analysis, we give some results of numerical calculation by using the Matlab procedure.

## 1. Introduction

In this paper, our purpose is to present and discuss a mixed finite element method for the time fractional telegraph equation

(1) ∂0,t2αu(x,t)+2κ∂0,tαu(x,t)−Δu(x,t)+βu(x,t) =f(x,t), (x,t)∈Ω×J,

with boundary condition

(2) $$\begin{array}{c} u\left(x,t\right)=0,\;\left(x,t\right)\in \partial \Omega \times \bar{J}, \end{array}$$

and initial conditions

(3) $$\begin{array}{c} u\left(x,0\right)=u_{0}\left(x\right),\;u_{t}\left(x,0\right)=u_{1}\left(x\right),\;x\in \Omega , \end{array}$$

where *Ω*(⊂*R* ^*d*^, *d* = 1,2, 3) is a bounded domain with boundary ∂*Ω* and *J* = (0, *T*] is the time interval with 0 < *T* < *∞*. The coefficients *κ* > 0 and *β* ≥ 0 are two constants, *f*(*x*, *t*) is a given source function, *u*_0_(*x*) and *u*_1_(*x*) are two given initial functions, and the time Caputo fractional-order derivatives ∂_0,*t*_^*α*^ *u*(*x*, *t*) and ∂_0,*t*_^2*α*^ *u*(*x*, *t*) are defined, respectively, by

(4) ∂0,tαu(x,t)=1Γ(1−α)∫0t∂u(x,τ)∂τdτ(t−τ)α,∂0,t2αu(x,t)=1Γ(2−2α)∫0t∂2u(x,τ)∂τ2dτ(t−τ)2α−1,

where 1/2 < *α* < 1.

In the current literatures, we can see that some numerical methods for solving fractional partial differential equations (PDEs), which include finite element methods [1–8], mixed finite element methods [9], finite difference methods [10–24], finite volume methods [25, 26], spectral methods [27], and discontinuous Galerkin methods [28–31], have been considered and analyzed. In 2014, Liu et al. [9] gave some theoretical error analysis for a class of fractional PDE based on a nonstandard mixed method in spatial direction and a finite difference scheme in time direction. In [32], Zhao and Li discussed finite element method for the fractional telegraph equation. In 2014, Wei et al. [31] studied the numerical solution for time fractional telegraph equation based on the LDG method. But, we have not seen any related studies on mixed finite element methods for solving the fractional telegraph equation.

Recently, some people have made use of the method to obtain the numerical solution for some partial differential equations since Pani (in 1998) [33] proposed an *H* ^1^-GMFE method. This method includes some advantages, such as avoiding the LBB consistency condition, allowing different polynomial degrees of the finite element spaces, and obtaining the optimal a priori estimates in both *H* ^1^ and *L* ^2^-norms. In [34], Pani and Fairweather discussed some detailed a priori error results of two numerical schemes based on the *H* ^1^-GMFE method for linear parabolic integrodifferential equations. In [35], Pani et al. gave some error analysis based on *H* ^1^-GMFE scheme for the partial differential equation of hyperbolic type. At the same time, a modified *H* ^1^-GMFE procedure was also proposed and analyzed for the case of two or three dimensions. Guo and Chen [36, 37] obtained some theoretical error analysis and numerical results of *H* ^1^-GMFE method for RLW equation and Sobolev equation. In 2013, Liu et al. [38] introduced another auxiliary variable, which is different from the one in [36], and then proposed and studied an explicit multistep *H* ^1^-GMFE scheme for a RLW equation. Liu and Li [39] and Zhou [40] gave some different discussions on *H* ^1^-GMFE method for pseudohyperbolic equations (heat transport equation), respectively. In [41], Che et al. studied the *H* ^1^-GMFE method for a nonlinear integrodifferential equation. Recently, Shi et al. [42, 43] proposed some nonconforming mixed scheme based on the *H* ^1^-GMFE method.

Based on the above review on *H* ^1^-GMFE method, we easily see that the method was studied based on the integer-order partial differential equations. However, the theoretical results of *H* ^1^-GMFE method for fractional telegraph equation have not been presented and analyzed.

In this paper, our aim is to give some detailed a priori error analysis and numerical results on the *H* ^1^-GMFE method for time fractional telegraph equation. We apply the difference schemes to approximate the time fractional derivatives and use the *H* ^1^-GMFE method to discretize spatial direction. We obtain some optimal a priori error results of one dimension for the scalar unknown in *L* ^2^ and *H* ^1^-norms. Moreover, we also get an a priori error result of the optimal *L* ^2^-norm for the auxiliary variable. At the same time, we use the *H* ^1^-GMFE method to deal with the cases in several dimensions and analyze the stable results for the *H* ^1^-GMFE scheme. We calculate some numerical results to verify the theoretical analysis of *H* ^1^-GMFE method for fractional telegraph equation.

The layout of the paper is as follows. In Section 2, we formulate an *H* ^1^-GMFE scheme for time fractional telegraph equation (5). In Section 3, we introduce some lemmas of two important projections and two difference approximations for time fractional derivatives and then analyze some a priori error results. In Section 4, some theoretical results are given in the cases of two and three dimensions. In Section 5, we choose a numerical example to verify the theoretical analysis of our method. In Section 6, we give some remarks and extensions about the *H* ^1^-GMFE method for fractional PDEs.

For the need of study, we denote the natural inner product as (·, ·) in (*L* ^2^(*Ω*))^*d*^, *d* = 1,2, 3. Further, we write the classical* Sobolev* spaces *W* ^*m*,2^(*Ω*) as *H* ^*m*^ with norm ||*z*||_*m*_ = [∑_0≤|*β*|≤*m*_∫_*Ω*_ | *D* ^*β*^ *z*|^2^ *dx*]^1/2^. When *m* = 0, we simply write the norm ||·||_0_ as ||·||.

## 2. An *H* ^1^-GMFE Scheme in One Space Dimension

In this section, we first consider the *H* ^1^-GMFE method for the following time fractional telegraph equation in 1D case:

(5) ∂0,t2αu(x,t)+2κ∂0,tαu(x,t)−∂2u(x,t)∂x2+βu(x,t) =f(x,t), (x,t)∈Ω×J,

with boundary condition

(6) $$\begin{array}{c} u\left(x_{L},t\right)=u\left(x_{R},t\right)=0,\;t\in \bar{J}, \end{array}$$

and initial conditions

(7) $$\begin{array}{c} u\left(x,0\right)=u_{0}\left(x\right),\;u_{t}\left(x,0\right)=u_{1}\left(x\right),\;x\in \Omega , \end{array}$$

where *Ω* = [*x*_*L*_, *x*_*R*_] ⊂ *R*.

In order to get the *H* ^1^-GMFE formulation, we first introduce an auxiliary variable *σ* = ∂*u*(*x*, *t*)/∂*x* and split (5) into the following first-order system by

(8) ∂0,t2αu(x,t)+2κ∂0,tαu(x,t)−∂σ(x,t)∂x  +βu(x,t)=f(x,t),

(9) $$\begin{array}{c} \sigma -\frac{\partial u\left(x,t\right)}{\partial x}=0. \end{array}$$

Multiplying (8) by −∂*w*/∂*x*, *w* ∈ *H* ^1^, integrating with respect to space from *x*_*L*_ to *x*_*R*_, and using an integration by parts with ∂*u*(*x*_*L*_, *t*)/∂*t* = ∂*u*(*x*_*R*_, *t*)/∂*t* = 0 and ∂^2^ *u*(*x*_*L*_, *t*)/∂*t* ^2^ = ∂^2^ *u*(*x*_*R*_, *t*)/∂*t* ^2^ = 0, we easily get

(10) (∂0,t2ασ,w)+2κ(∂0,tασ,w)+(∂σ∂x,∂w∂x) =β(u,∂w∂x)−(f,∂w∂x).

Multiply (9) by ∂*v*/∂*x*, *v* ∈ *H*_0_^1^, and integrate with respect to space from *x*_*L*_ to *x*_*R*_ to obtain

(11) $$\begin{array}{c} \left(\frac{\partial u}{\partial x},\frac{\partial v}{\partial x}\right)=\left(\sigma ,\frac{\partial v}{\partial x}\right),\;\forall v\in H_{0}^{1}. \end{array}$$

For formulating finite element scheme, we now choose the finite element spaces *V*_*h*_ ⊂ *H*_0_^1^ and *W*_*h*_ ⊂ *H* ^1^, which satisfy the following approximation properties: for 1 ≤ *p* ≤ *∞* and *k*, *r* positive integers [33],

(12) inf⁡vh∈Vh{||v−vh||Lp+h||v−vh||W1,p}  ≤Chk+1||v||Wk+1,p, v∈H01∩Wk+1,p,inf⁡wh∈Wh{||w−wh||Lp+h||w−wh||W1,p}  ≤Chr+1||w||Wr+1,p, w∈Wr+1,p.

Based on the chosen finite element spaces, the semidiscrete *H* ^1^-GMFE scheme is described by

(13) (∂uh∂x,∂vh∂x)=(σh,∂vh∂x), ∀vh∈Vh,(∂0,t2ασh,wh)+2κ(∂0,tασh,wh)+(∂σh∂x,∂wh∂x) =β(uh,∂wh∂x)−(f,∂wh∂x), ∀wh∈Wh.

## 3. Full Discrete Scheme and A Priori Error Estimates

### 3.1. Two Projection Lemmas

For a priori error estimates for fully discrete scheme, we introduce two projection operators [33, 44] in Lemmas 1 and 2.

**Lemma 1 .**

One defines a Ritz projection *P*_*h*_ *u* ∈ *V*_*h*_ for the variable *u* by

(14) $$\begin{array}{c} \left(u_{x}-P_{h}u_{x},v_{hx}\right)=0,\;v_{h}\in V_{h}. \end{array}$$

Then the following estimates hold, for *j* = 0,1:

(15) $$\begin{array}{c} {||u-P_{h}u||}_{j}\leq C_{⋆}h^{k+1-j}{||u||}_{k+1}. \end{array}$$

**Lemma 2 .**

Further, one also defines an elliptic projection *R*_*h*_ *σ* ∈ *W*_*h*_ of *σ* as the solution of

(16) $$\begin{array}{c} B\left(\sigma -R_{h}\sigma ,w_{h}\right)=0,\;w_{h}\in W_{h}, \end{array}$$

where *B*(*σ*, *w*) = (*σ*_*x*_, *w*_*x*_) + *λ*(*σ*, *w*). Here *λ* > 0 is chosen to satisfy

(17) $$\begin{array}{c} B\left(w,w\right)\geq \mu_{0}{||w||}_{1}^{2},\;w\in H^{1},\;\;\mu_{0}>0. \end{array}$$

Then the following estimates are found: for *j* = 0,1,

(18) $$\begin{array}{c} {||\sigma -R_{h}\sigma ||}_{j}\leq C_{*}h^{r+1-j}{||\sigma ||}_{r+1}. \end{array}$$

### 3.2. Approximation of Time-Fractional Derivative

For formulating fully discrete scheme, let 0 = *t*_0_ < *t*_1_ < *t*_2_ < ⋯<*t*_*M*_ = *T* be a given partition of the time interval [0, *T*] with step length Δ*t* = *T*/*M* and nodes *t*_*n*_ = *n*Δ*t*, for some positive integer *M*. For a smooth function *ϕ* on [0, *T*], define *ϕ* ^*n*^ = *ϕ*(*t*_*n*_). In the following analysis, for deriving the convenience of theoretical process, we now denote

(19) $$\begin{array}{c} B_{n-k}^{j\alpha }={\left(n-k+1\right)}^{j-j\alpha }-{\left(n-k\right)}^{j-j\alpha },\;j=1,2\\ D_{t}\sigma^{k}=\frac{\sigma^{k}-\sigma^{k-1}}{\Delta t},\;\;D_{tt}\sigma^{k-1}=\frac{\sigma^{k}-2\sigma^{k-1}+\sigma^{k-2}}{\Delta t^{2}}. \end{array}$$

Now, we will introduce two lemmas on the approximations of time fractional derivatives.

**Lemma 3 (see [27]).**

The time fractional derivative ∂_0,*t*_^*α*^ *σ*(*x*, *t*) at *t* = *t*_*n*_ is approximated by, for 0 < *α* < 1,

(20) $$\begin{array}{c} \partial_{0,t}^{\alpha }\sigma \left(x,t_{n}\right)=\frac{\Delta t^{1-\alpha }}{\Gamma \left(2-\alpha \right)}\overset{n}{\underset{k=1}{\sum }}B_{n-k}^{\alpha }D_{t}\sigma^{k}+E_{\alpha }^{n}, \end{array}$$

and then holds

(21) $$\begin{array}{c} \left|E_{\alpha }^{n}\right|\leq C_{0}\Delta t^{2-\alpha }. \end{array}$$

**Lemma 4 (see [1]).**

The time fractional order derivative ∂_0,*t*_^2*α*^ *σ*(*x*, *t*) at *t* = *t*_*n*_ is estimated by, for 1 < 2*α* < 2,

(22) $$\begin{array}{c} \partial_{0,t}^{2\alpha }\sigma \left(x,t_{n}\right)=\frac{\Delta t^{2-2\alpha }}{\Gamma \left(3-2\alpha \right)}\overset{n}{\underset{k=1}{\sum }}B_{n-k}^{2\alpha }D_{tt}\sigma^{k-1}+E_{2\alpha }^{n}, \end{array}$$

and then holds

(23) $$\begin{array}{c} \left|E_{2\alpha }^{n}\right|\leq C_{0}\Delta t. \end{array}$$

In the next analysis, we will derive and prove some a priori error results for *u* and *σ*.

### 3.3. Error Estimates for Fully Discrete Scheme

Based on the approximation formulas (20) and (22) of time-fractional derivatives, we obtain the time semidiscrete scheme of (10) and (11):

(24) (∂un∂x,∂v∂x)=(σn,∂v∂x), ∀v∈H01,Δt2−2αΓ(3−2α)∑k=1nBn−k2α(Dttσk−1,w)  +2κΔt1−αΓ(2−α)∑k=1nBn−kα(Dtσk,w)+(∂σn∂x,∂w∂x) =β(un,∂w∂x)−(fn,∂w∂x)+(E0n,w), ∀w∈H1,

where *E*_0_^*n*^ = *E*_*α*_^*n*^ + *E*_2*α*_^*n*^.

Now, we formulate a fully discrete procedure: find (*u*_*h*_^*n*^, *σ*_*h*_^*n*^) ∈ *V*_*h*_ × *W*_*h*_(*n* = 1,…, *M* − 1) such that

(25) (∂uhn∂x,∂vh∂x)=(σhn,∂vh∂x), ∀vh∈Vh,Δt2−2αΓ(3−2α)∑k=1nBn−k2α(Dttσhk−1,wh)  +2κΔt1−αΓ(2−α)∑k=1nBn−kα(Dtσhk,wh)+(∂σhn∂x,∂wh∂x) =β(uhn,∂wh∂x)−(fn,∂wh∂x), ∀wh∈Wh.

Making a combination of (24)-(25) with two projections (14) and (16), we get the following error equations:

(26) $$\begin{array}{c} \left(\frac{\partial \vartheta^{n}}{\partial x},\frac{\partial v_{h}}{\partial x}\right)=\left(\delta^{n},\frac{\partial v_{h}}{\partial x}\right)+\left(\varrho^{n},\frac{\partial v_{h}}{\partial x}\right),\;\forall v_{h}\in V_{h}, \end{array}$$

(27) Δt2−2αΓ(3−2α)∑k=1nBn−k2α(Dttδk−1,wh)  +2κΔt1−αΓ(2−α)∑k=1nBn−kα(Dtδk,wh)+B(δn,wh) =−Δt2−2αΓ(3−2α)∑k=1nBn−k2α(Dttϱk−1,wh)  −2κΔt1−αΓ(2−α)∑k=1nBn−kα(Dtϱk,wh)  +λ(ϱn,wh)+(E0n,wh)+β(ϕn+ϑn,∂wh∂x),∀wh∈Wh,

where

(28) ϕn+ϑn≐(u(tn)−Phun)+(Phun−uhn)=u(tn)−uhn;ϱn+δn≐(σ(tn)−Rhσn)+(Rhσn−σhn)=σ(tn)−σhn.

In the following discussion, we will derive the proof for the fully discrete a priori error estimates.

**Theorem 5 .**

With *σ*_0_ = *u*_0*x*_ and *σ*_*t*_(0) = *u*_1*x*_, suppose that *u* ^*n*^ ∈ *H*_0_^1^∩*H* ^*k*+1^, *σ* ^*n*^ ∈ *H* ^*r*+1^, *σ*_*h*_^0^ = *R*_*h*_ *σ*(0), and *σ*_*ht*_(0) = *L*_*h*_ *σ*_*t*_(0), where *L*_*h*_ is the *L* ^2^ projection defined by (*w* − *L*_*h*_ *w*, *w*_*h*_) = 0, *w*_*h*_ ∈ *W*_*h*_. Then there exists a positive constant *C*(*u*, *σ*, *α*, *β*) free of space-time discrete parameters *h* and Δ*t* such that, for 1/2 < *α* < 1,

(29) ||σn−σhn|| ≤C(u,σ,α,β)T2α−12−2α(Δt1−2αhmin⁡⁡{k+1,r+1}+Δt3−2α)  +C(σ)hr+1,

(30) ||un−uhn||j ≤C(u,σ,α,β)T2α−12−2α(Δt1−2αhmin⁡⁡{k+1,r+1}+Δt3−2α)  +C(σ)hr+1+C(u)hk+1−j, j=0,1,

and for *α* → 1

(31) ||σn−σhn||≤C(u,σ,β)(Δt−1hmin⁡⁡{k+1,r+1}+Δt)+C(σ)hr+1,

(32) ||un−uhn||j≤C(u,σ,β)(Δt−1hmin⁡⁡{k+1,r+1}+Δt)+C(σ)hr+1+C(u)hk+1−j, j=0,1.

**Proof**

For the need of error analysis, we first consider the *L* ^2^-norm ||*ϑ* ^*n*^|| and the *H* ^1^-norm ||*ϑ* ^*n*^||_1_. Taking *v*_*h*_ = *ϑ* ^*n*^ in (26) and using Poincaré inequality based on the *H*_0_^1^-space and Cauchy-Schwarz inequality, we easily get

(33) $$\begin{array}{c} ||\vartheta^{n}||\leq {||\vartheta^{n}||}_{1}\leq C||\frac{\partial \vartheta^{n}}{\partial x}||\leq C\left(||\varrho^{n}||+||\delta^{n}||\right). \end{array}$$

In the next analysis, we will give the estimates of ||*δ* ^*n*^|| and ||*ϑ* ^*n*^|| in *L* ^2^-norm. Noting that

(34) $$\begin{array}{c} \overset{n}{\underset{k=1}{\sum }}B_{n-k}^{\alpha }D_{t}\delta^{k}=\overset{n-1}{\underset{k=0}{\sum }}B_{k}^{\alpha }D_{t}\delta^{n-k},\\ \overset{n}{\underset{k=1}{\sum }}B_{n-k}^{2\alpha }D_{tt}\delta^{k-1}=\overset{n-1}{\underset{k=0}{\sum }}B_{k}^{2\alpha }D_{tt}\delta^{n-k-1}, \end{array}$$

then (27) may be rewritten as

(35) Δt2−2αΓ(3−2α)∑k=0n−1Bk2α(Dttδn−k−1,wh)  +2κΔt1−αΓ(2−α)∑k=0n−1Bkα(Dtδn−k,wh)+B(δn,wh) =−Δt2−2αΓ(3−2α)∑k=1nBn−k2α(Dttϱk−1,wh)  −2κΔt1−αΓ(2−α)∑k=1nBn−kα(Dtϱk,wh)  +λ(ϱn,wh)+(E0n,wh)+β(ϕn+ϑn,∂wh∂x).

We take *w*_*h*_ = *δ* ^*n*^ in (35) and multiply by

(36) $$\begin{array}{c} \Gamma_{\alpha }≐\Gamma \left(3-2\alpha \right)\Gamma \left(2-\alpha \right)\Delta t^{2\alpha }, \end{array}$$

to arrive at

(37) Γ(2−α)∑k=0n−1Bk2α(δn−k−2δn−k−1+δn−k−2,δn)  +2κΓ(3−2α)Δtα∑k=0n−1Bkα(δn−k−δn−k−1,δn)  +ΓαB(δn,δn) =−Γ(2−α)∑k=0n−1Bk2α(ϱn−k−2ϱn−k−1+ϱn−k−2,δn)  −2κΓ(3−2α)Δtα∑k=0n−1Bkα(ϱn−k−ϱn−k−1,δn)  +λΓα(ϱn,δn)+Γα(E0n,δn)+βΓα(ϕn+ϑn,∂ϑn∂x).

By the simple calculation, we get the following equality:

(38) Γ(2−α)∑k=0n−1Bk2α(δn−k−2δn−k−1+δn−k−2,δn)  +2κΓ(3−2α)Δtα∑k=0n−1Bkα(δn−k−δn−k−1,δn) =(Γ(2−α)+2κΓ(3−2α)Δtα)||δn||2+Γ(2−α)  ×[(B12α−2B02α)(δn−1,δn)(∑k=1n−1)−Bn2α(δ0,δn)+Bn−12α(δ−1,δn)+(∑k=1n−1(Bk+12α−2Bk2α+Bk−12α)δn−k−1,δn)]  +2κΓ(3−2α)Δtα  ×[(∑k=1n−1(Bkα−Bk−1α)δn−k,δn)−Bn−1α(δ0,δn)].

By applying the similar process of calculation to (38), we use Cauchy-Schwarz inequality to get

(39) −Γ(2−α)∑k=0n−1Bk2α(ϱn−k−2ϱn−k−1+ϱn−k−2,δn)  −2κΓ(3−2α)Δtα∑k=0n−1Bkα(ϱn−k−ϱn−k−1,δn) ≤(Γ(2−α)+2κΓ(3−2α)Δtα)||ϱn||+Γ(2−α)  ×[(2B02α−B12α)||ϱn−1||+Bn2α||ϱ0||+Bn−12α||ϱ−1||∑k=1n−1+∑k=1n−1|Bk+12α−2Bk2α+Bk−12α|||ϱn−k−1||]||δn||  +2κΓ(3−2α)Δtα  ×[∑k=1n−1|Bkα−Bk−1α|||ϱn−k||+Bn−1α||ϱ0||]||δn||.

Noting (33), we use Cauchy-Schwarz inequality and Young inequality to have

(40) βΓα(ϕn+ϑn,∂ϑn∂x) ≤βΓα||ϕn||||∂ϑn∂x||+βΓα||ϑn||||∂ϑn∂x|| ≤C(β)Γα||ϕn||(||ϱn||+||δn||)  +C(β)Γα(||ϱn||+||δn||)2 ≤C(β)Γα(||ϕn||2+||ϱn||2)+C(β)Γα||δn||2.

Substitute (38), (39), and (40) into (37) and use Cauchy-Schwarz inequality to arrive at

(41) (Γ(2−α)+2κΓ(3−2α)Δtα−C(β)Γα)||δn||2  +ΓαB(δn,δn) ≤((Γ(2−α)+2κΓ(3−2α)Δtα)||ϱn||+Γ(2−α)[∑k=1n−1]×[(2B02α−B12α)(||δn−1||+||ϱn−1||)∑k=1n−1+Bn2α(||δ0||+||ϱ0||)+Bn−12α(||δ−1||+||ϱ−1||)+∑k=1n−1|Bk+12α−2Bk2α+Bk−12α|∑k=1n−1×(||δn−k−1||+||ϱn−k−1||)][∑k=1n−1]+λΓα||ϱn||+Γα||E0n||)||δn||  +2κΓ(3−2α)Δtα  ×[∑k=1n−1|Bkα−Bk−1α|(||δn−k||+||ϱn−k||)∑k=1n−1+Bn−1α(||δ0||+||ϱ0||)]||δn||  +C(β)Γα(||ϕn||2+||ϱn||2).

By an application of Young inequality, we get

(42) Γ(2−α)||δn||+ΓαB(δn,δn) ≤1Γ(2−α)  ×((Γ(2−α)+2κΓ(3−2α)Δtα)||ϱn||+Γ(2−α)[∑k=1n−1]×[(2B02α−B12α)(||δn−1||+||ϱn−1||)∑k=1n−1+Bn2α(||δ0||+||ϱ0||)+Bn−12α(||δ−1||+||ϱ−1||)+∑k=1n−1|Bk+12α−2Bk2α+Bk−12α|∑k=1n−1×(||δn−k−1||+||ϱn−k−1||)][∑k=1n−1]+λΓα||ϱn||+Γα||E0n||)  +2κΓ(3−2α)ΔtαΓ(2−α)  ×[∑k=1n−1|Bkα−Bk−1α|(||δn−k||+||ϱn−k||)∑k=1n−1+Bn−1α(||δ0||+||ϱ0||)]  +C(β)Γα(||ϕn||+||ϱn||).

Noting that *B*_*k*_^2*α*^ − *B*_*k*+1_^2*α*^ > 0, we arrive at

(43) ∑k=1n−1|Bk+12α−2Bk2α+Bk−12α| ≤∑k=1n−1(Bk2α−Bk+12α)+∑k=1n−1(Bk−12α−Bk2α) =B12α−Bn2α+B02α−Bn−12α.

At the same time, noting that *B*_*k*_^*α*^ − *B*_*k*+1_^*α*^ > 0, we use the similar method to have

(44) $$\begin{array}{c} \overset{n-1}{\underset{k=1}{\sum }}\left|B_{k}^{\alpha }-B_{k-1}^{\alpha }\right|=B_{0}^{\alpha }-B_{n-1}^{\alpha }. \end{array}$$

By a combination of (43) and (44) with (18) and noting that *B*_0_^*α*^ = *B*_0_^2*α*^ = 1, we arrive at

(45) 1Γ(2−α)[((Γ(2−α)+2κΓ(3−2α)Δtα)||ϱn||[∑k=1n−1]+Γ(2−α)×[(2B02α−B12α)||ϱn−1||∑k=1n−1+Bn2α||ϱ0||+Bn−12α||ϱ−1||+∑k=1n−1|Bk+12α−2Bk2α+Bk−12α|||ϱn−k−1||])+2κΓ(3−2α)Δtα×[∑k=1n−1|Bkα−Bk−1α|||ϱn−k||+Bn−1α||ϱ0||]([∑k=1n−1])+λΓα||ϱn||+Γα||E0n||]  +C(β)Γα(||ϕn||+||ϱn||) ≤CΓ(2−α)(Γ(2−α)+2κΓ(3−2α)Δtα+Γ(2−α)[(B02α)]×[(2B02α−B12α)+Bn2α+Bn−12α+B12α−Bn2α+B02α−Bn−12α]+2κΓ(3−2α)Δtα[(B02α)]×[B0α−Bn−1α+Bn−1α]+λΓα)hr+1  +CΓ(3−2α)Γ(2−α)Δt1+2α =CΓ(2−α)(Γ(2−α)+2κΓ(3−2α)Δtα)hr+1  +C(u,σ,β)Γα(hr+1+hk+1)  +CΓ(3−2α)Γ(2−α)Δt1+2α.

Substitute (45) into (42) and note that *μ*_0_||*δ* ^*n*^||_1_ ≤ *B*(*δ* ^*n*^, *δ* ^*n*^)/||*δ* ^*n*^||_1_ ≤ *B*(*δ* ^*n*^, *δ* ^*n*^)/||*δ* ^*n*^|| to get

(46) Γ(2−α)||δn||+μ0Γα||δn||1 ≤Γ(2−α)[(2B02α−B12α)||δn−1||∑k=1n−1+Bn2α||δ0||+Bn−12α||δ−1||+∑k=1n−1|Bk+12α−2Bk2α+Bk−12α|||δn−k−1||]  +2κΓ(3−2α)ΔtαΓ(2−α)  ×[∑k=1n−1|Bkα−Bk−1α|||δn−k||+Bn−1α||δ0||]  +CΓ(2−α)(Γ(2−α)+2κΓ(3−2α)Δtα)hr+1  +C(u,σ,β)Γα(hr+1+hk+1)  +CΓ(3−2α)Γ(2−α)Δt1+2α.

In the following discussion, we apply mathematical induction to obtain the error result

(47) $$\begin{array}{c} ||\delta^{n}||\leq \frac{C\left(u,\sigma ,\alpha ,\beta \right)}{B_{n-1}^{2\alpha }}\left(h^{k+1}+h^{r+1}+\Delta t^{2}+\Delta t^{1+2\alpha }\right). \end{array}$$

Take *n* = 1 in (46) and note that *B*_0_^2*α*^ = 1; it is easy to find that the following inequality holds:

(48) ||δ1||+Δtα||δ1||1 ≤C(u,σ,α,β)(hk+1+hr+1+Δt2+Δt1+2α) ≤C(u,σ,α,β)B02α(hk+1+hr+1+Δt2+Δt1+2α).

Assuming that the inequalities ||*δ* ^*j*^|| ≤ (*C*(*u*, *σ*, *α*, *β*)/*B*_*j*−1_^2*α*^)(*h* ^*k*+1^ + *h* ^*r*+1^ + Δ*t* ^2^ + Δ*t* ^1+2*α*^) hold for *j* = 1,2,…, *n* − 1, we now prove that the inequality (47) holds. Noting that 1/*B*_*k*_^*jα*^ < 1/*B*_*k*+1_^*jα*^, *j* = 1,2, and combining (43) and (44) with (46), we have

(49) ||δn||+Δtα||δn||1 ≤(Γ(2−α)[(2B02α−B12α)C(u,σ,α,β)Bn−12α∑k=1n−1×(hr+1+hk+1+Δt2+Δt1+2α)+Bn2α||δ0||+Bn−12α||δ−1||+∑k=1n−1|Bk+12α−2Bk2α+Bk−12α|×C(u,σ,α,β)Bn−k−22α∑k=1n−1×(hr+1+hk+1+Δt2+Δt1+2α)])  +2κΓ(3−2α)ΔtαΓ(2−α)  ×[∑k=1n−1|Bkα−Bk−1α|C(u,σ,α,β)Bn−k−12α∑k=1n−1×(hr+1+hk+1+Δt2+Δt1+2α)+Bn−1α||δ0||]  +CΓ(2−α)(Γ(2−α)+2κΓ(3−2α)Δtα)hr+1  +C(u,σ,β)Γα(hr+1+hk+1)  +CΓ(3−2α)Γ(2−α)Δt1+2α ≤C(u,σ,α,β)Bn−12α(hr+1+hk+1+Δt2+Δt1+2α).

Based on the process of mathematical induction, we claim that (47) holds.

For the need of the next proof, we have to estimate the term *n* ^1−2*α*^/*B*_*n*−1_^2*α*^. We now use Taylor formula to arrive at

(50) n1−2αBn−12α=n1−2αn2−2α−(n−1)2−2α=1n−(n−1)(1+1/(n−1))2α−1=(n−(n−1)[(1n−1)2]×[1+(2α−1)1n−1+(2α−1)(2α−2)2!(1n−1)2×(1+θ1n−1)2α−3(1n−1)2])−1=(2−2α+(2α−1)(2−2α)2!(1+θ1n−1)2α−3×(1+θ1n−1)2α−3(1n−1))−1<12−2α,

where 0 < *θ* < 1 and 1/2 < *α* < 1. Noting that (*n*Δ*t*)^2*α*−1^ ≤ *T* ^2*α*−1^ and (50), we have

(51) ||δn||+Δtα||δn||1 ≤C(u,σ,α,β)Bn−12α(hr+1+hk+1+Δt2+Δt1+2α) =n2α−1Δt2α−1Δt1−2αn1−2αC(u,σ,α,β)Bn−12α  ×(hr+1+hk+1+Δt2+Δt1+2α) ≤T2α−1Δt1−2α2−2αC(u,σ,α,β)Bn−12α  ×(hr+1+hk+1+Δt2+Δt1+2α) =C(u,σ,α,β)T2α−12−2α  ×(Δt1−2α(hr+1+hk+1)+Δt2+Δt3−2α).

Substitute (51) into (33) and use (18) to get

(52) ||ϑn||+||ϑn||1 ≤C(||ϱn||+||δn||) ≤C(u,σ,α,β)T2α−12−2α  ×(Δt1−2α(hr+1+hk+1)+Δt2+Δt3−2α)  +C(σ)hr+1.

By combining (51) and (18) with triangle inequality, the *L* ^2^-norm estimate ||*σ* ^*n*^ − *σ*_*h*_^*n*^|| is got. Similarly, the estimate ||*u* ^*n*^ − *u*_*h*_^*n*^|| in the *L* ^2^-norm and the estimate ||*u* ^*n*^−*u*_*h*_^*n*^||_1_ in the *H* ^1^-norm also are obtained by a combination of (52) and (15) with triangle inequality.

Now we mainly analyze the case *α* → 1. We can find that the error inequality (29) in this case has no meaning since the coefficient *C*(*u*, *σ*, *α*, *β*)/(2 − 2*α*) → *∞* as *α* → 1. So, we have to look for another error estimate's process. Noting the fact that *n*Δ*t* ≤ *T* (*n* = 1,2,…, *M*), we can obtain the following error inequality:

(53) $$\begin{array}{c} ||\delta^{n}||\leq C\left(u,\sigma ,\alpha ,\beta \right)n\Delta t\left(\Delta t^{1-2\alpha }\left(h^{r+1}+h^{k+1}\right)+\Delta t^{3-2\alpha }\right). \end{array}$$

Now we can use induction to prove the inequality (53). The detailed proof is similar to the above process of analysis, so we do not give the detailed proof again. Making a combination of (53) and (18) with triangle inequality, we can get the estimate (31). A similar discussion for (32) can also be made.

## 4. An *H* ^1^-GMFE Scheme for Several Spaces Variables

In this section, we consider (1) with two and three spaces variables.

Let **L** ^2^(*Ω*) = (*L* ^2^(*Ω*))^*d*^, (*d* = 2 or 3) with inner product and norm

(54) $$\begin{array}{c} \left(q,w\right)=\overset{d}{\underset{i=1}{\sum }}\left(q_{i},w_{i}\right),\;\;||w||={\left(\overset{d}{\underset{i=1}{\sum }}{||w_{i}||}^{2}\right)}^{1/2}. \end{array}$$

Further, let

(55) $$\begin{array}{c} W=H\left(div;\Omega \right)=\left\{w\in L^{2}\left(\Omega \right)\;|\;\nabla \cdot w\in L^{2}\left(\Omega \right)\right\} \end{array}$$

with norm

(56) $$\begin{array}{c} {||w||}_{H(div;\Omega )}={\left({||w||}^{2}+{||\nabla \cdot w||}^{2}\right)}^{1/2}. \end{array}$$

Taking **q** = ∇*u*, we use a similar process to the system (10) and (11) to get the *H* ^1^-Galerkin mixed weak formulation {*u*, **q**}:[0, *T*] ↦ *H*_0_^1^ × **W** by

(57) (∇u,∇v)=(q,∇v), ∀v∈H01,(∂0,t2αq,w)+2κ(∂0,tαq,w)+(∇·q,∇·w) =β(u,∇·w)−(f,∇·w), ∀w∈W.

The corresponding time semidiscrete system is defined by

(58) (∇un,∇v)=(qn,∇v), ∀v∈H01,Δt2−2αΓ(3−2α)∑k=1nBn−k2α(Dttqk−1,w)  +2κΔt1−αΓ(2−α)∑k=1nBn−kα(Dtqk,w)+(∇·qn,∇·w) =β(un,∇·w)−(fn,∇·w)+(R0n,w), ∀w∈W,

where **R**_0_^*n*^ = *O*(Δ*t*), which can be estimated by a similar process to *E*_0_^*n*^.

In order to get fully discrete mixed finite element scheme, we now choose the finite element spaces *V*_*h*_ ⊂ *H*_0_^1^ and *W*_*h*_ ⊂ **W**, which satisfy the following approximation properties: for 1 ≤ *p* ≤ *∞* and *k*, *r* positive integers [33],

(59) inf⁡vh∈Vh{||v−vh||+h||v−vh||1} ≤Chk+1||v||k+1, v∈H01∩Hk+1,inf⁡wh∈Wh{||w−wh||+h||w−wh||H(div;Ω)} ≤Chr+1||w||r+1, w∈Hr+1.

The fully discrete *H* ^1^-GMFE scheme is to find {*u*_*h*_^*n*^, **q**_*h*_^*n*^}:[0, *T*] ↦ *V*_*h*_ × **W**_*h*_ such that

(60) $$\begin{array}{c} \left(\nabla u_{h}^{n},\nabla v_{h}\right)=\left(q_{h}^{n},\nabla v_{h}\right),\;\forall v_{h}\in V_{h}, \end{array}$$

(61) Δt2−2αΓ(3−2α)∑k=1nBn−k2α(Dttqhk−1,wh)  +2κΔt1−αΓ(2−α)∑k=1nBn−kα(Dtqhk,wh)+(∇·qhn,∇·wh) =β(uhn,∇·wh)−(fn,∇·wh), ∀w∈Wh.

### 4.1. The Analysis of Stability

In the following discussion, we will give the stability for the system (60) and (61). First, we need to obtain an important lemma on two initial value conditions.

**Lemma 6 .**

With **q**_0_ = ∇*u*_0_ and **q**_*t*_(0) = ∇*u*_1_, the following inequality holds:

(62) $$\begin{array}{c} ||q_{h}^{-1}||\leq C\left({||u_{0}||}_{1}+\Delta t{||u_{1}||}_{1}\right). \end{array}$$

**Proof**

By a similar discussion as in [32], we have

(63) $$\begin{array}{c} ||q_{h}^{-1}||\leq C\left(||q_{0}||+\Delta t||q_{t}\left(0\right)||\right). \end{array}$$

Based on the given initial value conditions and (63), we arrive at

(64) ||qh−1||≤C(||∇u0||+Δt||∇u1||)≤C(||u0||1+Δt||u1||1).

**Theorem 7 .**

The following stable inequality for the system (60) and (61) holds:

(65) ||uhn||1+||qhn||+κΓα||∇·qhn|| ≤C(α)(Γαmax⁡0≤j≤n||fj||+||u0||1+Δt||u1||1).

**Proof**

In (60), we take *v*_*h*_ = ∇*u*_*h*_^*n*^ and use Cauchy-Schwarz inequality and Poincaré inequality to arrive at

(66) $$\begin{array}{c} ||u_{h}^{n}||\leq {||u_{h}^{n}||}_{1}\leq C||\nabla u_{h}^{n}||\leq C||q_{h}^{n}||. \end{array}$$

In (61), we choose **w**_*h*_ = **q**_*h*_^*n*^ and use Cauchy-Schwarz inequality, Young inequality, and (66) to get

(67) Δt2−2αΓ(3−2α)∑k=1nBn−k2α(Dttqhk−1,qhn)  +2κΔt1−αΓ(2−α)∑k=1nBn−kα(Dtqhk,qhn)+||∇·qhn||2 ≤β||uhn||||∇·qhn||+||fn||||∇·qhn|| ≤C||qhn||||∇·qhn||+||fn||||∇·qhn|| ≤C(||qhn||2+||fn||2)+12||∇·qhn||2.

By the simple simplification for (67), we easily get

(68) Δt2−2αΓ(3−2α)∑k=1nBn−k2α(Dttqhk−1,qhn)  +2κΔt1−αΓ(2−α)∑k=1nBn−kα(Dtqhk,qhn)+12||∇·qhn||2 ≤C(||qhn||2+||fn||2).

For the case *n* = 1 in (68), we multiply (36) to get easily

(69) Γ(2−α)B02α(qh1−2qh0+qh−1,qh1)  +2κΔtαΓ(3−2α)B0α(qh1−qh0,qh1)+κΓα||∇·qh1||2 ≤CΓα(||qh1||2+||f1||2).

Noting that *B*_0_^*α*^ = 1 = *B*_0_^2*α*^ and Lemma 6, we use Cauchy-Schwarz inequality to get

(70) (12Γ(2−α)+κΔtαΓ(3−2α)−CΓα)||qh1||2  +κΓα||∇·qh1||2 ≤CΓα||f1||2+C(α)(||qh−1||2+||qh0||2) ≤Γα||f1||2+C(α)(||u0||12+Δt2||u1||12) ≤C(α)(Γαmax⁡0≤j≤n||fj||+||u0||1+Δt||u1||1)2.

By the simple calculation for (70), we arrive at

(71) ||qh1||+κΓα||∇·qh1|| ≤C(α)(Γαmax⁡0≤j≤n||fj||+||u0||1+Δt||u1||1).

For the case *n* ≥ 1, using a similar process to the proof of Theorem 5 based on the mathematical induction, we can get

(72) ||qhn||+κΓα||∇·qhn|| ≤C(α)(Γαmax⁡0≤j≤n||fj||+||u0||1+Δt||u1||1).

By a combination of (72) and (66), we get the conclusion of Theorem 7.

### 4.2. A Priori Error Results

For deriving the a priori error analysis, we define the Ritz projection ${\tilde{u}}_{h}\in V_{h}$ by

(73) $$\begin{array}{c} \left(\nabla \left(u-{\tilde{u}}_{h}\right),\nabla v_{h}\right)=0,\;v_{h}\in V_{h}. \end{array}$$

Further, let ${\tilde{q}}_{h}\in W_{h}$ be the standard finite element interpolant of **q**.

Let $\rho =q-{\tilde{q}}_{h}$, $\eta =u-{\tilde{u}}_{h}$; see [33, 44]; we obtain

(74) $$\begin{array}{c} {||\eta ||}_{j}\leq Ch^{k+1-j}{||u||}_{k+1},\;j=0,1,\\ ||\rho ||+h{||\rho ||}_{H(div;\Omega )}\leq Ch^{r+1}{||q||}_{r+1}. \end{array}$$

Now, we can get the following theorem of a priori error estimates based on the above contents.

**Theorem 8 .**

With **q**_0_ = ∇*u*_0_, **q**_*t*_(0) = ∇*u*_1_, *u* ^*n*^ ∈ *H*_0_^1^∩*H* ^*k*+1^, and **q** ^*n*^ ∈ **H** ^*r*+1^, there exists a positive constant *C*(*u*, **q**, *α*, *β*) free of space-time meshes *h* and Δ*t* such that, for 1/2 < *α* < 1,

(75) ||qn−qhn||≤C(u,q,α,β)T2α−12−2α(Δt1−2αhmin⁡⁡{k+1,r}+Δt3−2α)+C(q)hr+1,||un−uhn||j≤C(u,q,α,β)T2α−12−2α(Δt1−2αhmin⁡⁡{k+1,r}+Δt3−2α)+C(q)hr+1+C(u)hk+1−j, j=0,1,

and for *α* → 1

(76) ||qn−qhn||≤C(u,q,β)(Δt−1hmin⁡⁡{k+1,r}+Δt)+C(q)hr+1,||un−uhn||j≤C(u,q,β)(Δt−1hmin⁡⁡{k+1,r}+Δt)+C(q)hr+1+C(u)hk+1−j, j=0,1.

**Proof**

We can use a similar proof as in Theorem 5 to get the conclusion of Theorem 8, so we do not discuss that again.

## 5. Some Numerical Results

Here, in order to show the numerical performances on the rate of convergence and a priori error estimates, we now choose *κ* = 1/2 and *β* = 0, the exact solution *u*(*x*, *t*) = *t* ^2+*α*^sin(2*πx*), for all (*x*, *t*)∈[0,1]×[0,1], and the determined source term *f*(*x*, *t*) by the exact solution in (5) and then calculate some numerical results by using Matlab procedure. In the numerical calculation, the time direction is approximated by finite difference schemes and the spatial direction is discretized by the *H* ^1^-GMFE method. Now, we divide interval [0,1] into equal-length intervals *e*_*i*_ = [*x*_*i*_, *x*_*i*+1_], 0 ≤ *i* ≤ *N* − 1. Now we choose the piecewise linear spaces *V*_*h*_ = span⁡{*φ*_1_, *φ*_2_,…, *φ*_*N*−1_} and *W*_*h*_ = span⁡{*ψ*_0_, *ψ*_1_,…, *ψ*_*N*_} with index *k* = *r* = 1. Let

(77) $$\begin{array}{c} u_{h}^{n}=\overset{N-1}{\underset{k=1}{\sum }}u_{k}\varphi_{k},\;\;\sigma_{h}^{n}=\overset{N}{\underset{k=0}{\sum }}\sigma_{k}\psi_{k}. \end{array}$$

Based on the above expressions (77), we can get the following equivalent algebraic equation of (25):

(78) [(a+b)E+F]σ→n =a∑k=1n−1(−Bn−k+12α+2Bn−k2α−Bn−k−12α)Eσ→k−1  +a(2B02α−B12α)Eσ→n−1+b∑k=1n(−Bn−k+1α+Bn−kα)Eσ→k−1  −aBn−12αEσ→−1−f→n,Gu→n=Hσ→n,

where *a* = Δ*t* ^−2*α*^/Γ(3 − 2*α*), *b* = Δ*t* ^−*α*^/Γ(2 − *α*), ${\vec{u}}^{n}={\left(u_{1}^{n},u_{2}^{n},\ldots ,u_{N-1}^{n}\right)}^{T}$, ${\vec{\sigma }}^{n}={\left(\sigma_{0}^{n},\sigma_{1}^{n},\ldots ,\sigma_{N-1}^{n},\sigma_{N}^{n}\right)}^{T}$, ${\vec{f}}^{n}={\left((f^{n},\psi_{0}),\ldots ,(f^{n},\psi_{N})\right)}^{T}$,

(79) E=(h3h6h62h3h6⋱⋱⋱h62h3h6h6h3)(n+1)×(n+1),F=(1h−1h−1h2h−1h⋱⋱⋱−1h2h−1h−1h1h)(n+1)×(n+1),G=(2h−1h−1h2h−1h⋱⋱⋱−1h2h−1h−1h2h)(n−1)×(n−1),H=(120−12⋱⋱⋱120−12)(n−1)×(n+1).

Based on the algebraic equation (78), we calculate and get the detailed numerical results listed in Tables 1–4.

In Table 1, we calculate the numerical results of a priori error results and orders of convergence in *L* ^2^-norm for both *u* and *σ* with a fixed time step length Δ*t* = 1/4000 and the changed spatial meshes *h*_1_ = 1/20, *h*_2_ = 1/40, and *h*_3_ = 1/80. From the calculated data, we can clearly see that the orders of convergence in *L* ^2^-norm keep unchanged with the changed *α* = 0.6,0.7,0.8,0.9, which confirm the optimal second-order convergence results of *H* ^1^-Galerkin mixed finite element method. Similarly, we obtain optimal first-order rates of convergence of *H* ^1^-norm for *u* and *σ* in Table 2.

In Table 3, we calculate the numerical results of a priori error results and orders of convergence in *L* ^2^-norm for both *u* and *σ* with different space-time step length *h* = 5Δ*t* = 1/*M*  (*M* = 20,40,80). From the calculated data, we can find that the rates of convergence, which are higher than the results *O*(Δ*t* ^3−2*α*^ + *h* ^2^) of theory, gradually decrease with the increased *α* (which is taken from 0.6 to 0.9 with interval 0.1). In Table 4, some first-order convergence results for both *u* and *σ* in *H* ^1^-norm, which are unchanged with the changed *α* = 0.6,0.7,0.8,0.9, are given.

In view of the above analysis on the numerical results, we now announce that the time fractional telegraph equation can be well solved by the *H* ^1^-GMFE method.

## 6. Some Concluding Remarks and Extensions

As far as we know, more and more people have proposed and analyzed a lot of numerical methods for fractional partial differential equations. However, the discussions on mixed finite element methods for solving fractional partial differential equations are fairly limited. The a priori error analysis of *H* ^1^-GMFE method for time fractional telegraph equation, especially, has not been made and discussed. In this paper, we give the detailed proof's process of the error analysis on *H* ^1^-GMFE method for time fractional telegraph equation. Further, we provide a numerical procedure to verify the theoretical results of the studied method.

In the near future, our aim is to study an *H* ^1^-Galerkin moving mixed finite element method, which is based on a combination of *H* ^1^-GMFE methods and moving finite element methods.

## Figures and Tables

**Table 1:** Spatial convergence results in *L* ^2^-norm with fixed Δ*t* = 1/4000 and changed *α*.

*L* ^2^-norm	*α*	$h_{1}=\frac{1}{20}$	$h_{2}=\frac{1}{40}$	$h_{3}=\frac{1}{80}$	Order $\left(\frac{h_{1}}{h_{2}}\right)$ *l* ^*l*^ ^*l*^ ^*l*^ ^*l*^	Order $\left(\frac{h_{2}}{h_{3}}\right)$ *g*_*g*__*g*__*g*__*g*_
||*u* − *u*_*h*_||	0.6	5.163644*E* − 03	1.280561*E* − 03	3.129308*E* − 04	2.013469	2.039022
	0.7	5.051593*E* − 03	1.251162*E* − 03	3.044436*E* − 04	2.013469	2.039022
	0.8	4.916797*E* − 03	1.215362*E* − 03	2.936933*E* − 04	2.016333	2.049004
	0.9	4.758728*E* − 03	1.172117*E* − 03	2.795694*E* − 04	2.021459	2.067839

||*σ* − *σ*_*h*_||	0.6	4.396500*E* − 03	1.122373*E* − 03	3.219747*E* − 04	1.969804	1.801533
	0.7	5.141035*E* − 03	1.312040*E* − 03	3.759918*E* − 04	1.970247	1.803038
	0.8	6.036700*E* − 03	1.543010*E* − 03	4.444122*E* − 04	1.968013	1.795777
	0.9	7.087004*E* − 03	1.822010*E* − 03	5.343041*E* − 04	1.959645	1.769798

**Table 2:** Spatial convergence results in *H* ^1^-norm with fixed Δ*t* = 1/4000 and changed *α*.

*H* ^1^-norm	*α*	$h_{1}=\frac{1}{20}$	$h_{2}=\frac{1}{40}$	$h_{3}=\frac{1}{80}$	Order $\left(\frac{h_{1}}{h_{2}}\right){{{l^{l}}^{l}}^{l}}^{l}$	Order $\left(\frac{h_{2}}{h_{3}}\right){{{g_{g}}_{g}}_{g}}_{g}$
	0.6	6.933461*E* − 01	3.484812*E* − 01	1.744206*E* − 01	0.992495	0.998510
||*u*−*u*_*h*_||_1_	0.7	6.933489*E* − 01	3.484816*E* − 01	1.744207*E* − 01	0.992499	0.998511
	0.8	6.933531*E* − 01	3.484823*E* − 01	1.744208*E* − 01	0.992505	0.998513
	0.9	6.933591*E* − 01	3.484833*E* − 01	1.744210*E* − 01	0.992513	0.998516
	0.6	4.374396*E* + 00	2.191164*E* + 00	1.096078*E* + 00	0.997386	0.999347
||*σ*−*σ*_*h*_||_1_	0.7	4.374658*E* + 00	2.191198*E* + 00	1.096083*E* + 00	0.997450	0.999363
	0.8	4.374979*E* + 00	2.191241*E* + 00	1.096089*E* + 00	0.997528	0.999383
	0.9	4.375365*E* + 00	2.191294*E* + 00	1.096098*E* + 00	0.997620	0.999406

**Table 3:** Space-time convergence results in *L* ^2^-norm with *h* = 5Δ*t* = 1/*M* and changed *α*.

*L* ^2^-norm	*α*	*M*_1_ = 20	*M*_2_ = 40	*M*_3_ = 80	Order $\left(\frac{M_{1}}{M_{2}}\right){{{l^{l}}^{l}}^{l}}^{l}$	Order $\left(\frac{M_{2}}{M_{3}}\right){{{g_{g}}_{g}}_{g}}_{g}$
||*u* − *u*_*h*_||	0.6	4.781196*E* − 03	1.095478*E* − 03	2.262375*E* − 04	2.125811	2.275651
	0.7	4.587916*E* − 03	1.028885*E* − 03	2.011905*E* − 04	2.156757	2.354448
	0.8	4.319590*E* − 03	9.315674*E* − 04	1.630376*E* − 04	2.213162	2.514455
	0.9	3.941100*E* − 03	7.841911*E* − 04	2.154685*E* − 04	2.329321	1.863729

||*σ* − *σ*_*h*_||	0.6	6.937717*E* − 03	2.316455*E* − 03	8.737355*E* − 04	1.582542	1.406650
	0.7	8.221980*E* − 03	2.746089*E* − 03	1.033148*E* − 03	1.582108	1.410332
	0.8	1.000490*E* − 02	3.373945*E* − 03	1.275972*E* − 03	1.568199	1.402840
	0.9	1.251982*E* − 02	4.324761*E* − 03	1.669151*E* − 03	1.533521	1.373505

**Table 4:** Space-time convergence results in *H* ^1^-norm with *h* = 5Δ*t* = 1/*M* and changed *α*.

*H* ^1^-norm	*α*	*M*_1_ = 20	*M*_2_ = 40	*M*_3_ = 80	Order $\left(\frac{M_{1}}{M_{2}}\right){{{l^{l}}^{l}}^{l}}^{l}$	Order $\left(\frac{M_{2}}{M_{3}}\right){{{g_{g}}_{g}}_{g}}_{g}$
||*u*−*u*_*h*_||_1_	0.6	6.933582*E* − 01	3.484856*E* − 01	1.744222*E* − 01	0.992502	0.998515
	0.7	6.933669*E* − 01	3.484880*E* − 01	1.744230*E* − 01	0.992510	0.998519
	0.8	6.933819*E* − 01	3.484924*E* − 01	1.744244*E* − 01	0.992523	0.998525
	0.9	6.934089*E* − 01	3.485010*E* − 01	1.744275*E* − 01	0.992543	0.998536

||*σ*−*σ*_*h*_||_1_	0.6	4.375309*E* + 00	2.191391*E* + 00	1.096134*E* + 00	0.997538	0.999422
	0.7	4.375792*E* + 00	2.191479*E* + 00	1.096153*E* + 00	0.997639	0.999456
	0.8	4.376484*E* + 00	2.191614*E* + 00	1.096183*E* + 00	0.997779	0.999505
	0.9	4.377505*E* + 00	2.191830*E* + 00	1.096235*E* + 00	0.997973	0.999579

## References

[1] Li C, Zhao Z, Chen Y (2011). Numerical approximation of nonlinear fractional differential equations with subdiffusion and superdiffusion. *Computers and Mathematics with Applications*.

[2] Sun H, Chen W, Sze KY (2013). A semi-discrete finite element method for a class of time-fractional diffusion equations. *Philosophical Transactions of the Royal Society of London A*.

[3] Jiang YJ, Ma JT (2011). High-order finite element methods for time-fractional partial differential equations. *Journal of Computational and Applied Mathematics*.

[4] Zheng YY, Li CP, Zhao ZG (2010). A note on the finite element method for the space-fractional advection diffusion equation. *Computers & Mathematics with Applications*.

[5] Ma JT, Liu JQ, Zhou ZQ (2014). Convergence analysis of moving finite element methods for space fractional differential equations. *Journal of Computational and Applied Mathematics*.

[6] Ford NJ, Xiao J, Yan Y (2011). A finite element method for time fractional partial differential equations. *Fractional Calculus and Applied Analysis*.

[7] Zhang N, Deng WH, Wu YJ (2012). Finite difference/element method for a two-dimensional modified fractional diffusion equation. *Advances in Applied Mathematics and Mechanics*.

[8] Zhao ZG, Zheng YY (2014). Leapfrog/finite element method for fractional diffusion equation. *The Scientific World Journal*.

[9] Liu Y, Li H, Gao W, He S, Fang ZC (2014). A new mixed finite element method for a class of time-fractional partial differential equations. *The Scientific World Journal*.

[10] Meerschaert MM, Tadjeran C (2004). Finite difference approximations for fractional advection-dispersion flow equations. *Journal of Computational and Applied Mathematics*.

[11] Atangana A, Baleanu D (2013). Numerical solution of a kind of fractional parabolic equations via two difference schemes. *Abstract and Applied Analysis*.

[12] Meerschaert MM, Tadjeran C (2006). Finite difference approximations for two-sided space-fractional partial differential equations. *Applied Numerical Mathematics*.

[13] Liu F, Zhuang P, Anh V, Turner I, Burrage K (2007). Stability and convergence of the difference methods for the space-time fractional advection-diffusion equation. *Applied Mathematics and Computation*.

[14] Zhang H, Liu F, Phanikumar MS, Meerschaert MM (2013). A novel numerical method for the time variable fractional order mobile-immobile advection-dispersion model. *Computers & Mathematics with Applications*.

[15] Baleanu D, Diethelm K, Scalas E, Trujillo JJ (2012). *Fractional Calculus: Models and Numerical Methods*.

[16] Shen S, Liu F, Anh V, Turner I, Chen J (2013). A characteristic difference method for the variable-order fractional advection-diffusion equation. *Journal of Applied Mathematics and Computing*.

[17] Wang K, Wang H (2011). A fast characteristic finite difference method for fractional advection-diffusion equations. *Advances in Water Resources*.

[18] Lin R, Liu F, Anh V, Turner I (2009). Stability and convergence of a new explicit finite-difference approximation for the variable-order nonlinear fractional diffusion equation. *Applied Mathematics and Computation*.

[19] Yuste SB, Quintana-Murillo J (2012). A finite difference method with non-uniform timesteps for fractional diffusion equations. *Computer Physics Communications*.

[20] Yuste SB (2006). Weighted average finite difference methods for fractional diffusion equations. *Journal of Computational Physics*.

[21] Sousa E (2012). A second order explicit finite difference method for the fractional advection diffusion equation. *Computers & Mathematics with Applications*.

[22] Sun H, Chen W, Li C, Chen Y (2012). Finite difference schemes for variable-order time fractional diffusion equation. *International Journal of Bifurcation and Chaos in Applied Sciences and Engineering*.

[23] Zhang YN, Sun ZZ, Zhao X (2012). Compact alternating direction implicit scheme for the two-dimensional fractional diffusion-wave equation. *SIAM Journal on Numerical Analysis*.

[24] Gao G-H, Sun Z-Z (2013). The finite difference approximation for a class of fractional sub-diffusion equations on a space unbounded domain. *Journal of Computational Physics*.

[25] Yang QQ, Turner I, Moroney T, Liu FW (2014). A finite volume scheme with preconditioned Lanczos method for two-dimensional space-fractional reaction-diffusion equations. *Applied Mathematical Modelling*.

[26] Liu F, Zhuang P, Turner I, Burrage K, Anh V (2013). A new fractional finite volume method for solving the fractional diffusion equation. *Applied Mathematical Modelling*.

[27] Lin YM, Xu CJ (2007). Finite difference/spectral approximations for the time-fractional diffusion equation. *Journal of Computational Physics*.

[28] Deng WH, Hesthaven JS Local discontinuous Galerkin methods for fractional ordinary differential equations. http://arxiv.org/abs/1403.5759.

[29] Ren Z, Wei L, He Y, Wang S (2012). Numerical analysis of an implicit fully discrete local discontinuous Galerkin method for the fractional Zakharov-Kuznetsov equation. *Mathematical Modelling and Analysis*.

[30] Zhang X, Liu J, Wen J, Tang B, He YN (2013). Analysis for one-dimensional time-fractional Tricomi-type equations by LDG methods. *Numerical Algorithms*.

[31] Wei L, Dai H, Zhang D, Si Z (2014). Fully discrete local discontinuous Galerkin method for solving the fractional telegraph equation. *Calcolo*.

[32] Zhao ZG, Li CP (2012). Fractional difference/finite element approximations for the time-space fractional telegraph equation. *Applied Mathematics and Computation*.

[33] Pani AK (1998). An *H*
^1^-Galerkin mixed finite element method for parabolic partial differential equations. *SIAM Journal on Numerical Analysis*.

[34] Pani AK, Fairweather G (2002). *H*
^1^-Galerkin mixed finite element methods for parabolic partial integro-differential equations. *IMA Journal of Numerical Analysis*.

[35] Pani AK, Sinha RK, Otta AK (2004). An *H*
^1^-Galerkin mixed method for second order hyperbolic equations. *International Journal of Numerical Analysis and Modeling*.

[36] Guo L, Chen HZ (2006). *H*
^1^-Galerkin mixed finite element metho d for the regularized long wave equation. *Computing*.

[37] Guo L, Chen HZ (2006). *H*
^1^-Galerkin mixed finite element method for the Sobolev equation. *Journal of Systems Science and Mathematical Sciences*.

[38] Liu Y, Li H, Du Y, Wang J (2013). Explicit multistep mixed finite element method for RLW equation. *Abstract and Applied Analysis*.

[39] Liu Y, Li H (2009). *H*
^1^-Galerkin mixed finite element methods for pseudo-hyperbolic equations. *Applied Mathematics and Computation*.

[40] Zhou Z (2010). An *H*
^1^-Galerkin mixed finite element method for a class of heat transport equations. *Applied Mathematical Modelling*.

[41] Che HT, Zhou ZJ, Jiang ZW, Wang YJ (2013). *H*
^1^-Galerkin expanded mixed finite element methods for nonlinear pseudo-parabolic integro-differential equations. *Numerical Methods for Partial Differential Equations*.

[42] Zhang YD, Shi DY (2013). Superconvergence of an *H*
^1^-Galerkin nonconforming mixed finite element method for a parabolic equation. *Computers & Mathematics with Applications*.

[43] Shi D, Tang Q (2013). Nonconforming *H*
^1^-Galerkin mixed finite element method for strongly damped wave equations. *Numerical Functional Analysis and Optimization*.

[44] Wheeler MF (1973). A priori *L*
_2_ error estimates for Galerkin approximations to parabolic partial differential equations. *SIAM Journal on Numerical Analysis*.

